# A nightmare of clopidogrel resistance in a resource-limited setting: case report of subacute stent thrombosis

**DOI:** 10.1186/s43044-023-00408-8

**Published:** 2023-10-12

**Authors:** Pedro Pallangyo, Smita V. Bhalia, Makrina Komba, Zabella S. Mkojera, Henry A. Mayala, Engerasiya Kifai, Peter R. Kisenge

**Affiliations:** 1 Department of Research and Training, Jakaya Kikwete Cardiac Institute, P.O Box 65141, Dar es Salaam, Tanzania; 2 Department of Cardiology, Jakaya Kikwete Cardiac Institute, P.O Box 65141, Dar es Salaam, Tanzania; 3 Department of Clinical Support Services, Jakaya Kikwete Cardiac Institute, P.O Box 65141, Dar es Salaam, Tanzania

**Keywords:** Stent thrombosis, Myocardial infarction, Acute coronary syndrome, Clopidogrel resistance, Percutaneous coronary intervention, Coronary artery disease

## Abstract

**Background:**

Stent thrombosis, a life-threatening complication of percutaneous coronary intervention (PCI) continues to occur despite effective antiplatelet regimens and improved stenting methods. Noncompliance with dual antiplatelet therapy is the most common etiology; however, in spite of timely and their optimum administration the rates of recurrent myocardial infarction (MI) and stent thrombosis remain high. Clopidogrel resistance is increasingly evoked with elevated risk of anterothrombotic events particularly in the setting of stent implantation. In this case report, we present a case of subacute stent thrombosis associated with clopidogrel resistance in a resource-constrained setting.

**Case presentation:**

A 60 year old man with a long standing history of hypertension presented with a 6-month history of progressive shortness of breath. Initial electrocardiogram (ECG) revealed T-wave inversion on lateral leads and echocardiogram revealed akinetic basal lateral wall and hypokinetic mid lateral wall with reduced systolic functions. An elective coronary angiography (CAG) revealed a 90% stenosis of mid left anterior descending (LAD) artery and an 80% stenosis on the proximal left circumflex artery. He underwent a successful PCI with a drug-eluting stent implantation to mid LAD. He was discharged in a stable state 48 h post revascularization with dual antiplatelet (clopidogrel and acetylsalicylic acid). Seven days later, he presented with a crushing substernal chest pain. Cardiac enzymes were elevated and ECG revealed anterior ST-elevation MI. An emergency CAG revealed a high thrombus burden with 100% occlusion of mid LAD. Following unsuccessful ballooning, intravenous and intracoronary thrombolysis with tenecteplase was given. A TIMI II flow was achieved and the patient was sent to the coronary care unit. However, 14 h later there was yet a new onset of severe chest pain. A 12-lead ECG previewed anterior ST-elevation MI and the cardiac enzymes were high. Urgent CAG revealed in-stent thrombotic total occlusion of mid LAD. A stent in stent was then implanted and TIMI III flow was restored. Clopidogrel resistance was suspected and the patient was transitioned to ticagrelol. There were no further ischemic events during the remainder of hospitalization and the patient was discharged in a hemodynamically stable state three days later. During follow-up after one and three months, he was fairly stable without any further cardiac events.

**Conclusions:**

Owing to clopidogrel resistance, stent thrombosis in the setting of dual antiplatelet therapy compliance may occur. While in a situation of clopidogrel resistance newer and more potent antiplatelet drugs should be used, their availability and cost remains a significant barrier particularly in the developing world. Nonetheless, a high index of suspicion and timely revascularization is fundamental to restore patency of the thrombosed vessel and confer better risk-adjusted survival rates.

## Background

Since Jacques Puel implanted the first stainless steel stent in 1986, the dramatic evolution of stents has incrementally improved prognosis following percutaneous coronary intervention (PCI) and adamantly revolutionized the management of coronary artery disease (CAD) [1–6]. Considering the aforementioned procedural success and the rising frequency of angiographic procedures globally, the focus has now evolved to stent thrombosis and delayed re-endothelialization. Stent thrombosis, an infrequent (incidence < 2%) [7–12] yet life-threatening complication of PCI continues to occur despite effective antiplatelet regimens and improved stenting methods. Moreover, stent thrombosis which often necessitate emergency revascularization is associated with high morbidity and mortality (up to 50%) [13–20].

Dual antiplatelet therapy with acetylsalicylic acid (ASA) and clopidogrel is the cornerstone of modern pharmacotherapy for preventing aberrant platelet activation in patients undergoing coronary angioplasty with stent implantation [21–27]. However, in spite of timely and optimum administration of antiplatelet drugs the rates of recurrent myocardial infarction (MI) and stent thrombosis remain high 28, 29. With a prevalence ranging between 4 and 44%, clopidogrel resistance is increasingly evoked with elevated risk of anterothrombotic events particularly in the setting of stent implantation [30–34]. In this case report, we present a case of subacute stent thrombosis associated with clopidogrel resistance in a resource-constrained setting.

## Case presentation

**Fig. 1:**
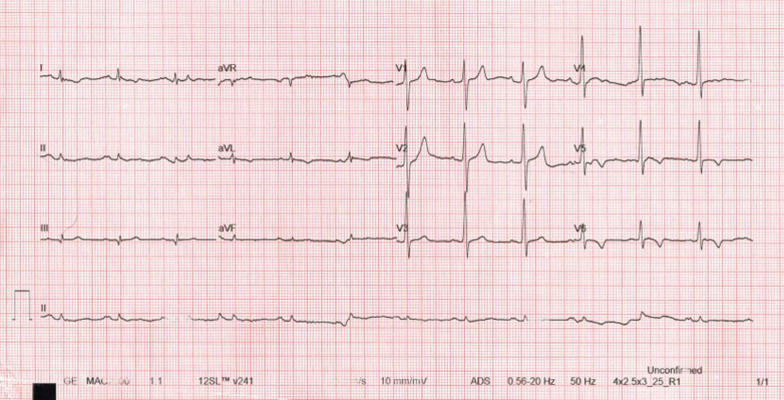
ECG displaying sinus rhythm with T-wave inversion on lateral leads

**Fig. 2:**
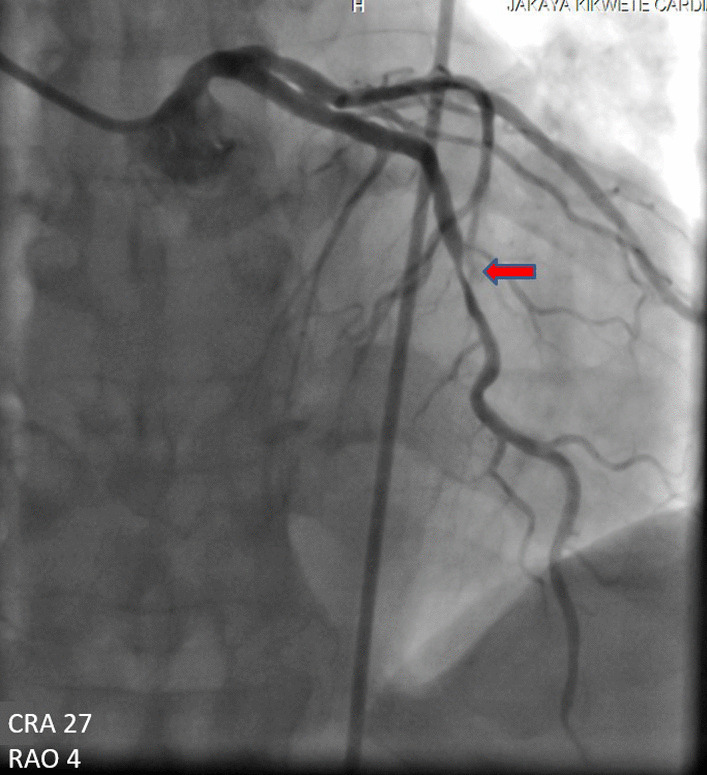
CAG showing a 90% stenosis of mid LAD artery

**Fig. 3:**
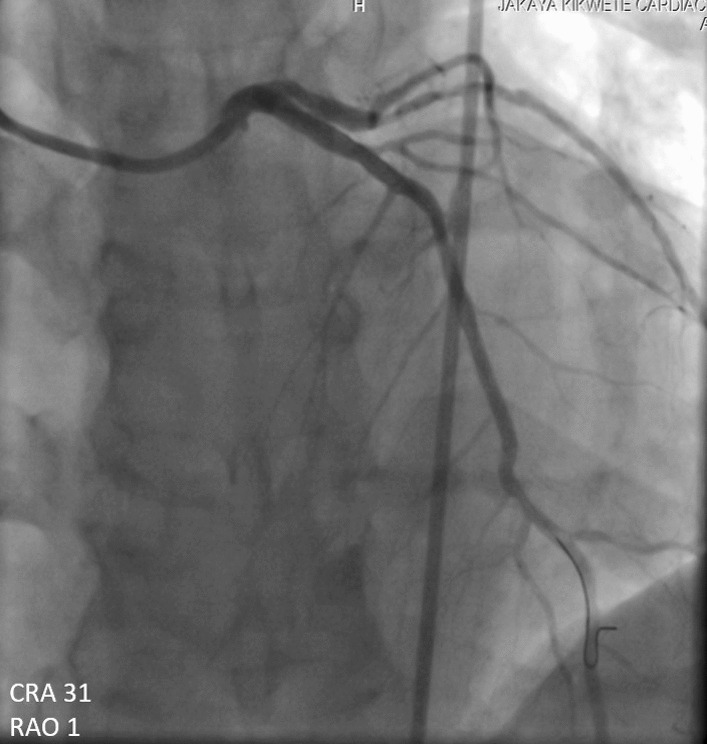
CAG showing successful revascularization post DES implantation

A 60-year-old man of African origin from Northern Tanzania presented with a 6-month history of progressive shortness of breath (SOB). He had a 13-years history of systemic hypertension; however, he denied a family history of premature coronary artery disease (CAD). Troponin-I was 1.8 ng/ml, initial electrocardiogram (ECG) revealed T-wave inversion on lateral leads (Fig. 1) and echocardiogram (ECHO) revealed akinetic basal lateral wall and hypokinetic mid lateral wall with reduced systolic functions (EF 45%). We reached a diagnosis of NSTEMI. An elective coronary angiography (CAG) revealed a 90% stenosis of mid left anterior descending (LAD) artery (Fig. 2) and an 80% stenosis on the proximal left circumflex (LCx) artery. He received a loading dose of clopidogrel (300 mg) and ASA (300 mg) and subsequently underwent a successful PCI with a drug-eluting stent (DES) implantation to mid LAD, Fig. 3. He lodged in the coronary care unit (CCU) for 48 h and was discharged in a stable state with clopidogrel 75 mg, ASA 75 mg, Telmisartan 80 mg, Metoprolol 50 mg and Atorvastatin 40 mg.

**Fig. 4:**
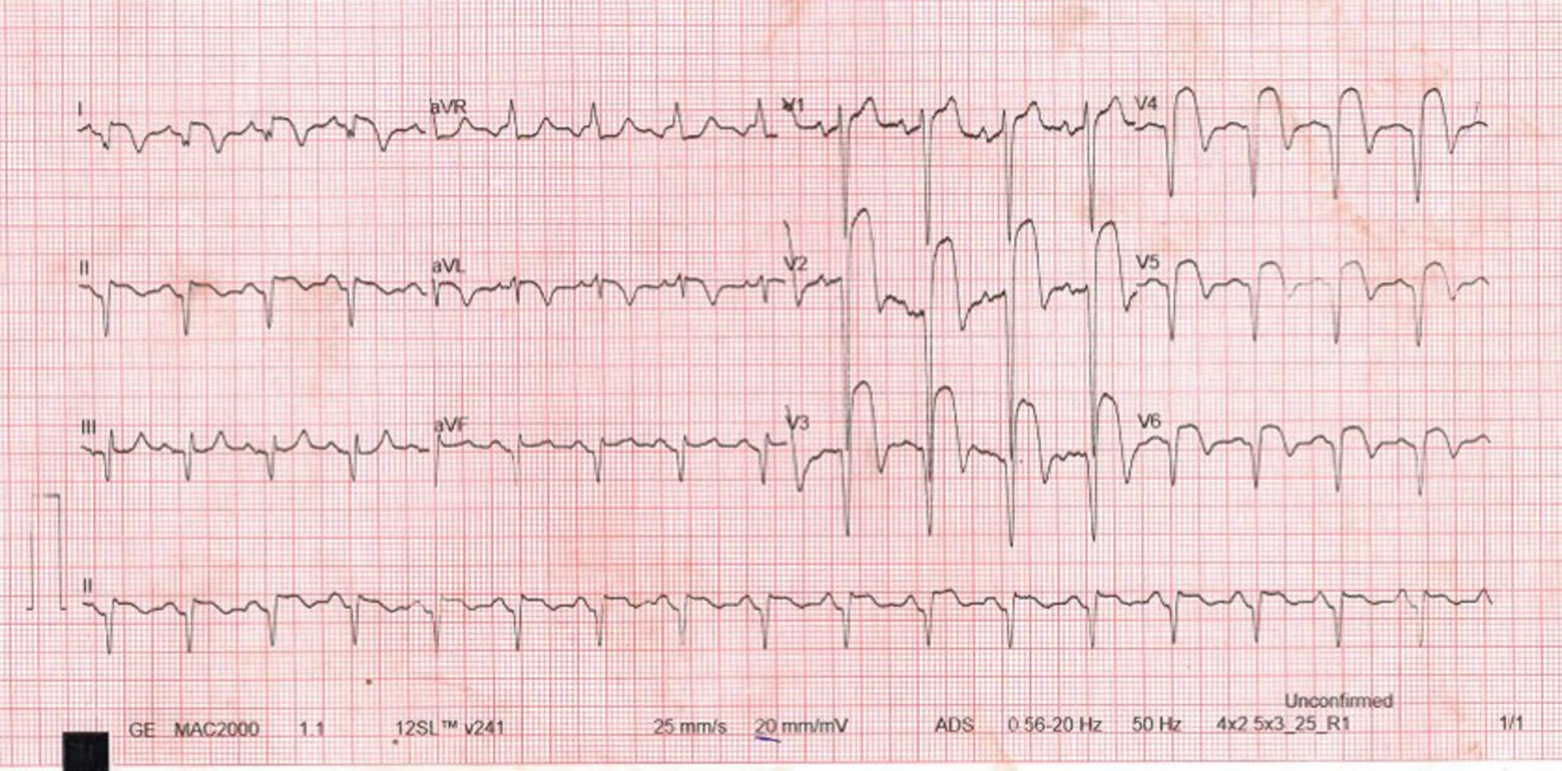
ECG displaying ST-elevation on anterolateral leads with Q-waves on anterolateral and inferior leads

**Fig. 5:**
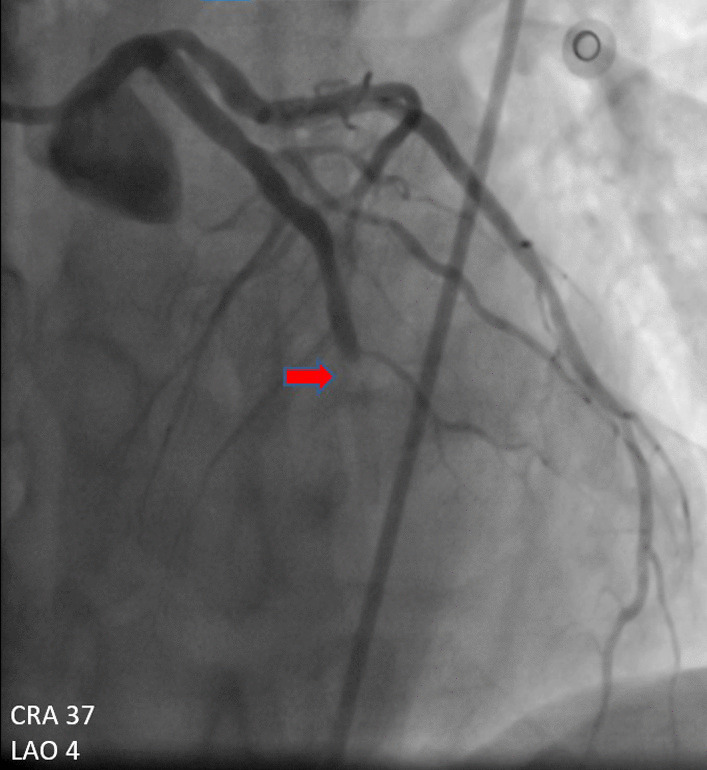
CAG showing stent thrombosis with total occlusion of mid LAD artery

**Fig. 6:**
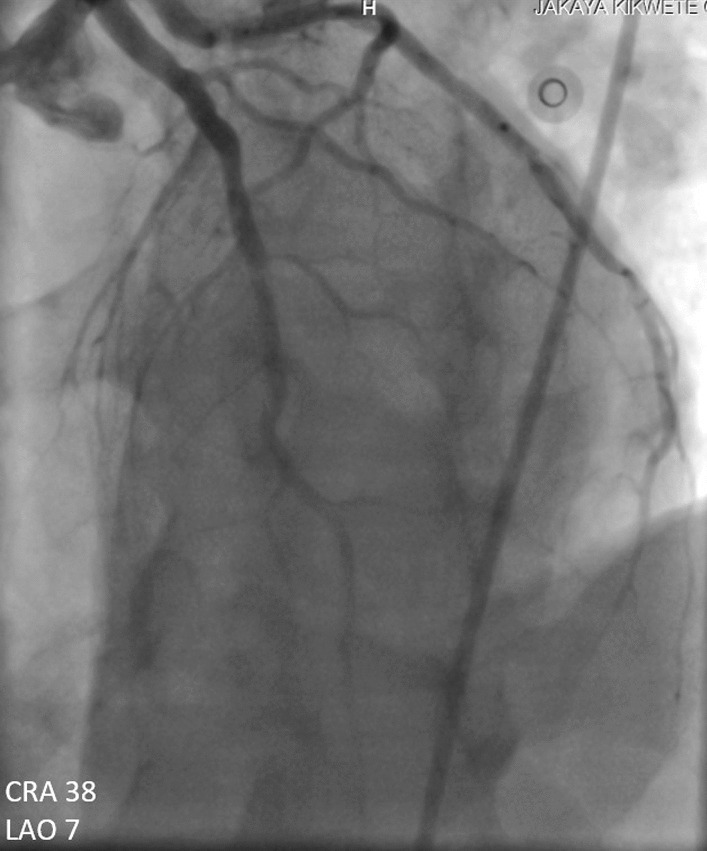
CAG post thrombolysis showing TIMI II flow patency

**Fig. 7:**
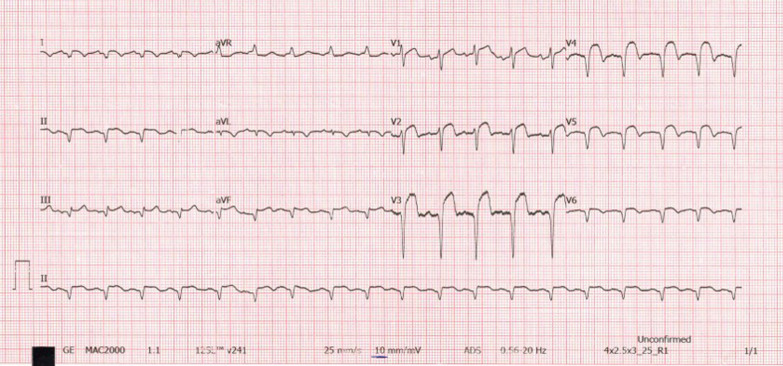
ECG displaying slight reduction in ST-segment amplitude on anterolateral leads with Q-waves on anterolateral & inferior leads

He presented seven days following discharge with a crushing substernal chest pain and ECG at this point showed anterior STEMI, Fig. 4. The patient was compliant with his treatment (including dual antiplatelet therapy). The peak level of creatine kinase-myoglobin binding (CK-MB) was 280 ng/ml and troponin-I was 52 ng/ml. An emergency CAG was performed and it revealed a high thrombus burden with 100% occlusion of mid LAD, Fig. 5. A two-wire technique was deployed to cross the lesion; however, ballooning was unsuccessful. Glycoprotein IIb/IIIa inhibitors are unavailable in the whole of East Africa region; however, an intracoronary thrombolysis with 15 mg of tenecteplase coupled with 25 mg IV dosage was given. Within a span of five minutes, a thrombolysis in myocardial infarction (TIMI) II flow was achieved (Figs. 6 and 7) and the patient was sent to the CCU.

**Fig. 8:**
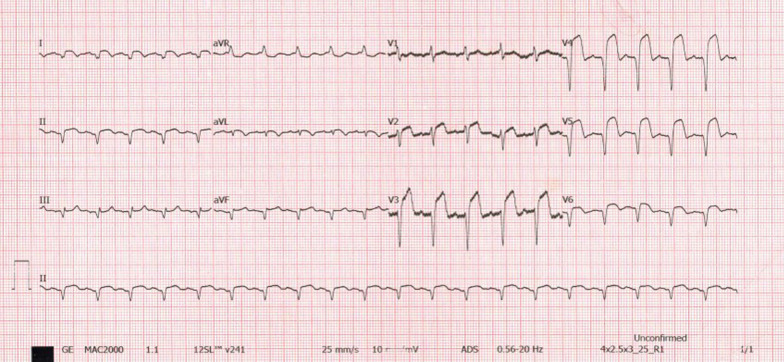
ECG displaying increase in ST-segment amplitude on anterolateral leads with Q-waves on anterolateral & inferior leads

**Fig. 9:**
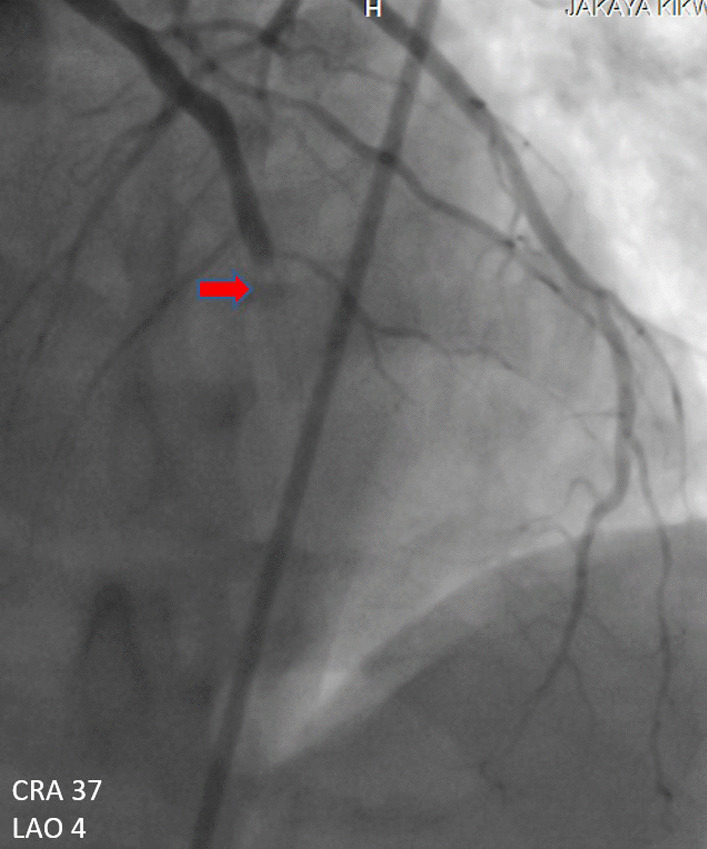
CAG showing stent thrombosis with total occlusion of mid LAD artery

**Fig. 10:**
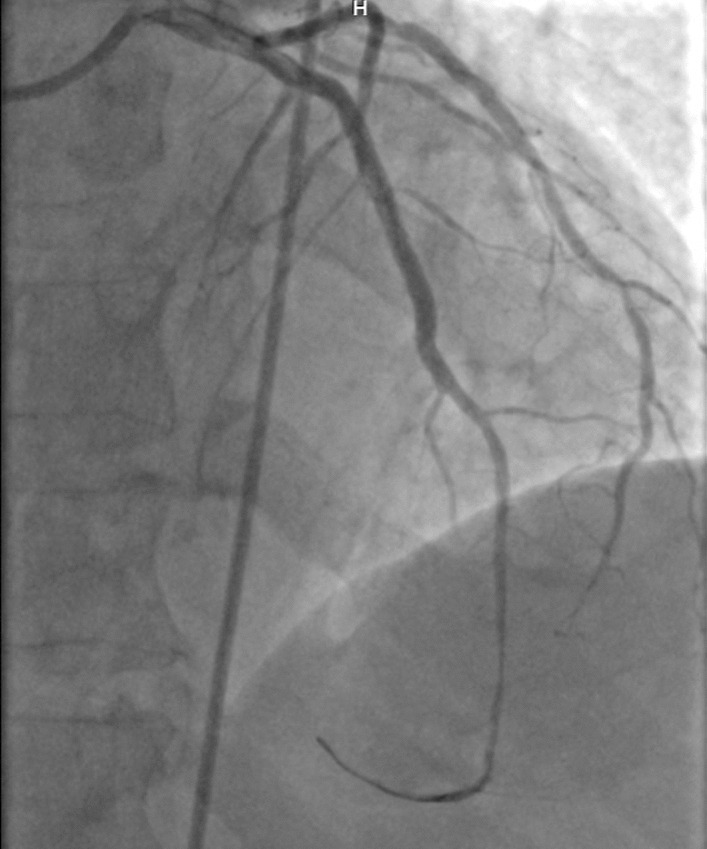
CAG showing successful revascularization (TIMI III flow) following stent in stent

**Fig. 11:**
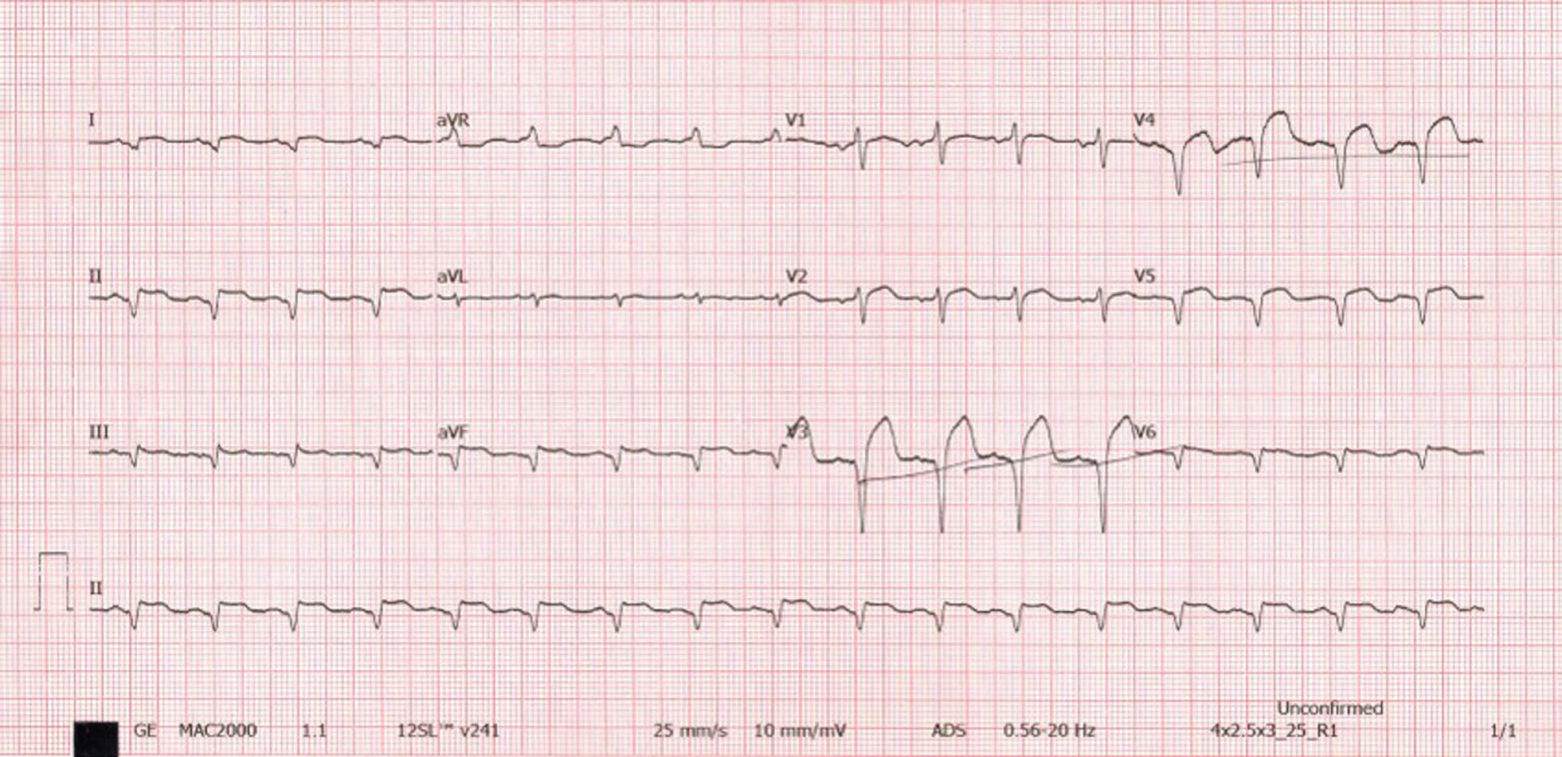
ECG showing significant reduction in ST-segment amplitude on anterolateral leads with Q-waves on the anterolateral & inferior leads

However, 14 h later there was yet a new onset of severe chest pain. A 12-lead ECG previewed anterior STEMI (Fig. 8) and the levels of CK-MB and troponin-I were 300 ng/ml and 88 ng/ml respectively. Urgent CAG revealed in-stent thrombotic total occlusion of mid LAD, Fig. 9. A stent in stent was then implanted and TIMI III flow was restored, Figs. 10 and 11. Clopidogrel resistance was suspected and as per the recommended guidelines, we transitioned the patient to a more potent antiplatelet drug (i.e., ticagrelol 90 mg). There were no further ischemic events during the remainder of hospitalization and the patient was discharged in a hemodynamically stable state three days later. At present, the National Insurance policy in Tanzania does not allow revascularization of non-infarct-related (non-IRA) vessel in the same admission (i.e., of IRA). Nonetheless, four weeks later, he successfully underwent PCI with DES to proximal LCx with desirable angiographic outcomes. During follow-up after one, three and six months, he was adherent to ticagrelol, remained symptom-free and repeat ECHO (at 6 months) showed improved LV systolic functions (i.e., EF 58%) with limited residual basal lateral and mid lateral wall hypokinesia.

## Discussion

Acute coronary syndromes (ACS) and attributable complications remain one of the most daunting challenges facing the medical fraternity across the globe. However, PCI, the commonest therapeutic procedure in contemporary medical practice has significantly improved clinical outcomes and survival prospects of ACS patients worldwide [1–6]. In conjunction with ASA, clinical trials have demonstrated significant relative risk reduction in MI, subacute stent thrombosis and ischemic death among patients treated with clopidogrel [21–27]. Nonetheless, owing to genetic polymorphism, conventional doses of clopidogrel exhibits high inter-individual variability in platelet aggregation inhibition potentially increasing the risk of stent thrombosis. [30–35]

Noncompliance with dual antiplatelet therapy is the most frequent cause of stent thrombosis; however, other factors including inadequate stent deployment, stent malapposition and unrecognized coronary dissection are usually implicated [16–20]. Repeat PCI is the most practiced approach in managing stent thrombosis. Current guidelines do not recommend the routine use of thrombectomy during PCI [36], and as witnessed in this case, despite of a high thrombus burden aspiration was not attempted. Moreover, intravascular imaging-guided PCI optimizes stent and patient-oriented outcomes (i.e., associated with improved survival and lower MI) compared to conventional angiography-guided PCI. Unfortunately intravascular imaging with either intravascular ultrasound [IVUS] or optical coherence tomography [OCT] techniques are unavailable in Tanzania and most of the Sub-Saharan Africa region. Nonetheless, diagnosing clopidogrel resistance could be challenging and diagnosis is often based on clinical grounds [37]. Despite being relatively inexpensive ($25) and pretty rapid (< 10 min) [34], tests to assess for high platelet reactivity (HPR) on clopidogrel could not be performed in our setting due to their unavailability. Even though credible diagnostic stratification remains a challenge, there is no convincing evidence to support routine assessment of clopidogrel resistance in the setting of coronary angioplasty [38, 39].

Similar to many other countries in the SSA region, none of the newer antiplatelet agents (i.e., ticagrelol and prasugrel) is currently licensed in Tanzania and although ticagrelol is limitedly sold in Dar es Salaam, the current cost of $236 for 30 tablets is unbearably high to most people living in such impoverished settings. With the current state where newer antiplatelet drugs are not licensed (i.e., their availability not guaranteed) and relatively expensive (i.e., currently not covered by national health insurance), patients like this one remain at high risk of recurrent stent thrombosis and sudden cardiac death. Furthermore, with the increasing catheterizations in the developing world, unavailability and high cost of newer antiplatelet drugs particularly in the setting of clopidogrel resistance leads to overutilization of scarce medical resources and undermines the revascularization efforts. Moreover, as a result of the extreme scarcity of interventional cardiology facilities in SSA amidst the sharp rise of ischemic heart disease (IHD) [40], such setbacks continues to deter attainment of the sustainable development goals (SDGs) in the region.

## Conclusions

Owing to clopidogrel resistance, stent thrombosis in the setting of dual antiplatelet therapy compliance may occur. While preprocedural testing for clopidogrel resistance might be of value, there is insufficient evidence to support routine screening in clinical practice. Moreover, in a situation of clopidogrel resistance newer and more potent antiplatelet drugs should be used; however, their availability and cost remains a significant barrier particularly in the developing world. Nonetheless, a high clinical suspicion and prompt revascularization measures are fundamental to restore patency of the thrombosed vessel and confer better risk-adjusted survival rates.

## Data Availability

The authors confirm that the data supporting the findings of this study are available within the article and its additional files.

## Abbreviations

ACS: Acute coronary syndrome
ASA: Acetylsalicylic acid
CAG: Coronary angiography
CAD: Coronary artery disease
CCU: Coronary care unit
CK-MB: Creatine kinase-myoglobin binding
DES: Drug-eluting stent
ECHO: Echocardiogram
EF: Ejection fraction
ECG: Electrocardiogram
HPR: High platelet reactivity
IHD: Ischemic heart disease
IRA: Infarct-related artery
IVUS: Intravascular ultrasound
LAD: Left anterior descending
LCx: Left circumflex
MI: Myocardial infarction
OCT: Optical coherence tomography
PCI: Percutaneous coronary intervention
SDGs: Sustainable development goals
SOB: Shortness of breath
SSA: Sub-Saharan Africa
STEMI: ST-elevation myocardial infarction
TIMI: Thrombolysis in myocardial infarction
